# The role of sirtuins in Alzheimer's disease

**DOI:** 10.3389/fnagi.2013.00016

**Published:** 2013-04-09

**Authors:** Rakhee Lalla, Gizem Donmez

**Affiliations:** Department of Neuroscience, Tufts University School of MedicineBoston, MA, USA

**Keywords:** sirtuins, aging, Alzheimer's disease, neurodegeneration, genetic models

## Abstract

Sirtuins are highly conserved NAD^+^-dependent enzymes that were shown to have beneficial effects against age-related diseases. Alzheimer's Disease (AD) is the most common neurodegenerative disorder associated with aging and the effects of sirtuins on AD have been investigated using different mouse and cell culture models. In most of these studies, it has been found that the overexpression of SIRT1 has protective effects against the AD phenotype. Therefore, designing therapeutics based on SIRT1 activity might be important to investigate treatment methods for this disease. In this review, we summarize the recent research regarding the functions of sirtuins and their potential roles in designing therapeutics for AD.

## Introduction

Sirtuins are conserved NAD^+^-dependent enzymes that display beneficial effects in age-related disorders. They are also stress-response proteins that help mammals adapt to dietary manipulations (Donmez and Guarente, 2010). Various substrates have been discovered for sirtuins, especially for SIRT1, the most widely studied sirtuin in the brain (Haigis and Sinclair, 2010). SIRT1 has been analysed in many common neurodegenerative disorders such as Alzheimer's, Parkinson's, and Huntington's Diseases, among others (Donmez, 2012). In this review, we will focus on the findings regarding the functions of sirtuins and their implications in Alzheimer's Disease (AD).

### Sirtuin biology

Sirtuins were first identified in yeast, named as silent information regulators (SIRs) and grouped as class III histone deacetylases (HDACs). They function by removing acetyl groups from lysines via consuming NAD (Sinclair and Guarente, 1997). There are seven human homologs of sirtuins (SIRT1-7), each of which displays different enzymatic activities and functions (Table 1). SIRT1, 2, and 3 have strong deacetylase activities, while SIRT4, 5, and 6 have display weak deacetylase activities. SIRT4 and SIRT6 also display ADP-ribosylase activities, and SIRT5 was shown to have demalonylase and desuccinylase activities (Shih and Donmez, 2013). In addition to having unique functions, sirtuins are also located in different areas of the cell, including the nucleus (SIRT1, 6 and 7), cytoplasm (SIRT2), and mitochondria (SIRT3-5) (Table 1). SIRT1, however, was also reported to shuttle between cytoplasm and nucleus (Imai and Guarente, 2010).

**Table 1:** **Activity and localization of mammalian sirtuins and their effects on AD**.

	**Enzymatic activity**	**Sub-cellular localization**	**Effects on AD**	**Targets related to AD**
SIRT1	Deacetylase	Nuclear, cytoplasmic	Neuroprotective (Chen et al., 2005; Qin et al., 2006b; Kim et al., 2007; Julien et al., 2009; Donmez et al., 2010; Min et al., 2010)	RARb, Tau, PGC1a, LXR, NFkB
SIRT2	Deacetylase	Cytoplasmic, nuclear	Genetic association (Polito et al., 2012)	Unknown
SIRT3	Deacetylase	Mitochondrial	Genetic association (Weir et al., 2012)	Unknown
SIRT4	Deacetylase ADP-ribosyltransferase	Mitochondrial	Unknown	Unknown
SIRT5	Deacetylase Demalonylase Desuccinylase	Mitochondrial	Unknown	Unknown
SIRT6	ADP-ribosyltransferase Deacetylase	Nuclear	Unknown	Unknown
SIRT7	Deacetylase	Nucleolar	Unknown	Unknown

Numerous target proteins and enzymes have been identified for sirtuins located in the nucleus, cytoplasm, or mitochondria (Haigis and Sinclair, 2010). It is established that NAD-dependent post-translational modification of these targets by sirtuins leads to the regulation of important biological processes in different subcellular compartments and also the effects on age-related disorders (Donmez and Guarente, 2010).

### Alzheimer's disease

AD is the most common neurodegenerative condition and the most common form of dementia in the elderly. Unfortunately, there is no cure for this disease. In 2006, there were 26.6 million people who suffered from AD worldwide, and the disease is predicted to affect 1 in 85 people globally by 2050 (Brookmeyer et al., 2007). Patients with AD suffer from neurological dysfunction resulting in memory loss, as well as cognitive and functional decline. AD is identified neuropathologically in mouse models and postmortem patient brains by the presence of amyloid plaques and neurofibrillary tangles. In addition, dominant mutations were found in the Amyloid Precursor Protein (APP) gene and in the presenilin 1 and 2 genes (PSEN1 and PSEN2), which encode for components of gamma-secretase (Hardy and Selkoe, 2002). APP is cleaved by beta-secretase and gamma-secretase sequentially to generate Abeta 1-40 and Abeta 1-42 amyloid peptides, which accumulate to form the amyloid plaques that are characteristic of AD (Tanzi and Bertram, 2005).

Neurofibrillary tangles are aggregates made up of hyperphosphorylated microtubule-associated protein tau. The tau proteins are the product of alternative splicing from a single gene that is designated as MAPT (microtubule-associated protein tau) in humans (Weingarten et al., 1975). All of the six tau isoforms are present in a hyperphosphorylated state as filaments in the AD brain. These tau aggregates were also reported to be present in other neurodegenerative disorders (Alonso et al., 2001).

## Role of SIRT1 in AD

### Initial studies

SIRT1 is the only sirtuin that has been extensively studied in AD. Previous studies showed that the adaptive responses to calorie restriction (CR) are mediated by SIRT1 (Imai and Guarente, 2010). SIRT1-deficient mice were reported not to be able to adapt to CR normally (Boily et al., 2008). On the other hand, the phenotypes of SIRT1-overexpressing transgenic mice mimic the physiological changes observed in CR (Bordone et al., 2007). These studies established the connection between CR and SIRT1. The effect of CR on AD was first observed in studies where the Abeta plaques were reduced in the brains of AD mice that are calorie-restricted (Patel et al., 2005). Additionally, the cortex of calorie-restricted squirrel monkeys displayed a reduction in Abeta plaques, which was inversely correlated with SIRT1 levels (Qin et al., 2006a).

In a following study, activation of SIRT1 was also shown to be the mechanism underlying prevention of amyloid neuropathology by CR in mouse (Qin et al., 2006b). The authors showed that exogenous human SIRT1 resulted in reduced serine/threonine Rho kinase ROCK1 expression and elevated alpha-secretase activity *in vivo*. ROCK1 inhibits non-amyloidogenic alpha-secretase processing of APP (Qin et al., 2006b). These results demonstrated SIRT1 as an underlying mechanism through which CR rescues the AD phenotype *in vivo*.

### Studies with cell culture models

SIRT1 was found to be protective against stress in a study conducted with cultured neuronal cells (Qin et al., 2006b). In this study, SIRT1 expression was found to promote alpha-secretase activity and attenuates Abeta peptides in primary Tg2576 neuron cultures and CHO-APPswe cells. In another study, SIRT1 was also shown to protect against microglia-dependent amyloid-beta toxicity in cells through inhibiting inflammatory NF-kappaB signaling (Chen et al., 2005). In addition to these, N2A cells stably overexpressing APPswe/PSEN1dE9 transgenes transfected with SIRT1 displayed reduced Abeta peptides and increased ADAM10 protein levels (Donmez et al., 2010).

Cell culture studies have also helped to discover the effect of SIRT1 in the tau model and inducible p25 models of AD. In a recent study, SIRT1 was found to deacetylate tau in HEK293T cells and primary cortical neurons during maturation in culture (Min et al., 2010). An earlier study showed that treatment of primary cortical neurons with low concentrations of ionomycin and H_2_O_2_ induces rapid upregulation of SIRT1 associated with generation of p25 (Kim et al., 2007). The authors showed that, in cell-based models for AD/tauopathies and amyotrophic lateral sclerosis (ALS), SIRT1 and resveratrol, a SIRT1-activating molecule (STAC), promote neuronal survival (Kim et al., 2007).

### Studies with genetic mouse models

One of the first studies that showed SIRT1 protects against AD and ALS used mouse models and primary neurons challenged with neurotoxic insults (Kim et al., 2007). The authors showed that resveratrol reduced neurodegeneration in the hippocampus, prevented learning impairment, and decreased the acetylation of the known SIRT1 substrates PGC-1alpha and p53 in an inducible p25 transgenic mouse, a model of AD and tauopathies (Kim et al., 2007). In addition, increased levels of SIRT1 in primary neurons were shown to confer protection against neurotoxicity induced by a mutant form of superoxide dismutase 1 (SOD1G37R), which has been linked to human ALS (Kim et al., 2007).

Other studies showed that SIRT1 targets both Abeta and tau *in vivo*. By crossing a SIRT1-overexpressing mouse or a SIRT1 brain-specific knockout mouse with the APPswe, PSEN1dE9 mouse model, SIRT1 expression could be elevated or deleted in the mouse brain, respectively (Donmez et al., 2010). The authors observed that overexpression of SIRT1 reduces Abeta plaques in the mouse brain, while deletion of SIRT1 exacerbates it. As an underlying mechanism, SIRT1 was shown to deacetylate retinoic acid receptor (RAR)-beta and activates ADAM10 (alpha-secretase) transcription, which increases ADAM10 levels in neurons and leads to upregulated APP processing by alpha-secretase (Donmez et al., 2010).

In addition to reducing Abeta formation, SIRT1 also inhibits the tau-related AD phenotype. In one study, SIRT1 reduction was found to parallel tau accumulation (Min et al., 2010). SIRT1 decreases tau accumulation by deacetylating the acetylated tau and consequently reducing its level. Conversely, SIRT1 inhibition leads to the opposite effect, increasing levels of tau, and exacerbating the accumulation of pathogenic forms of phosphorylated tau (Min et al., 2010).

One study used triple-transgenic mouse model of AD, which is known to accumulate amyloid beta and tau (Julien et al., 2009). Cortical SIRT1 RNA and protein levels were analyzed in this mouse model and found to remain unchanged (Julien et al., 2009).

### Patient-related studies

Having identified the potential protective role of SIRT1 in cell culture and genetic mouse models, it is also crucial to investigate the association of SIRT1 with the disease in AD patients. A recent study compared the mRNA and protein expression levels of SIRT1 in the brains of AD patients and controls using Western immunoblots and *in situ* hybridization (Julien et al., 2009). A significant reduction in RNA and protein expression levels of SIRT1 was reported in the parietal cortex of AD patients. This reduction was not observed in the cerebellum. The authors conducted further analyses with a second cohort of 36 subjects demonstrating that cortical SIRT1 was decreased in individuals with AD but not with mild cognitive impairment. SIRT1 mRNA and protein levels were negatively correlated with the duration of symptoms and the accumulation of tau, but were weakly correlated with the amyloid-beta 42. The authors concluded that the results of the study indicate that the loss of SIRT1 is closely associated with the accumulation of amyloid-beta and tau in the cerebral cortex of AD patients (Julien et al., 2009).

### Role of other sirtuins in AD

Not much is known about the role of SIRT3 in AD. One recent study showed that pharmacological augmentation of mitochondrial ROS increases Sirt3 expression in primary hippocampal culture (Weir et al., 2012). Furthermore, SIRT3 mRNA is upregulated in a specific spatio-temporal manner in a PDAPP mouse model of AD, which overexpresses human APP carrying the V717F mutation and forms Abeta plaques in mouse brain. In this study, Sirt3 mRNA expression is significantly increased in samples of the AD temporal cortex compared to matched controls (Weir et al., 2012).

SIRT2 has also been found to be associated with AD, as demonstrated by a genetic case-control study (Polito et al., 2012). In this study, three single nucleotide polymorphisms (SNPs) were analysed in a number of AD patients and non-demented control subjects. An association between SIRT2 rs10410544 T allele and AD was found in the APOE *ε*4-negative Caucasian population, necessitating further investigation (Polito et al., 2012). In the same study, authors also identified three SNPs for the SIRT3 gene.

## Conclusions

Although, SIRT2 and SIRT3 seem to play a role in AD based on recent research, SIRT1 is the most extensively studied sirtuin in AD. Interestingly, overexpression of SIRT1 was found to be protective in most of the studies conducted. This might lead to the design of SIRT1 activators that are able to cross the blood brain barrier to treat AD. Obviously, SIRT1 has numerous targets, which is a concern since activating various targets might not be beneficial for a specific disease. Overall, studies that use animal and cell culture models show that SIRT1 plays a neuroprotective role in the brain, a finding that encourages the design of therapeutics for AD based on SIRT1 activation.

Resveratrol is a natural polypenolic compound that is known as an activator of SIRT1. However, the association between SIRT1 and the mechanisms and the biological effects of resveratrol is currently debated. Three STACs, SRT1460, SRT1720, and SRT2183, which are structurally unrelated to, and 1000-fold more potent than resveratrol, have also been identified as a result of high throughput screens. When these activators were implemented in rodent animal studies, including high-fat-diet-induced obese mice, ob/ob mice, and Zucker fa/fa rats, they were able to normalize glucose homeostasis (Milne et al., 2007). A very recent study showed that specific hydrophobic motifs found in SIRT1 substrates such as PGC-1α and FOXO3a facilitate SIRT1 activation by STACs (Hubbard et al., 2013). The authors also demonstrated that a single amino acid in SIRT1, Glu(230), located in a structured N-terminal domain, is critical for activation by all previously reported STAC scaffolds and a new class of chemically distinct activators. It was concluded therefore, that SIRT1 can be directly activated through an allosteric mechanism common to chemically diverse STACs (Hubbard et al., 2013).

Nicotinamide (NAM) inhibits the catalytic activity of sirtuins by binding to a conserved region in the catalytic site (Avalos et al., 2005). The first sirtuin inhibitor, sirtinol was identified by phenotypic screening in yeast (Grozinger et al., 2001) and was shown to act as an anticancer molecule in various models, inhibiting Ras-MAPK pathway (Ota et al., 2006).

In future studies, it would be interesting to see whether or not other sirtuins have an effect on the AD phenotype or disease pathology. Except for SIRT1, none of the sirtuins have been analysed extensively in AD. Studying the other sirtuins will also unravel their novel functions in the mammalian brain. Identifying these functions of sirtuins and their relations to AD will give rise to therapeutic avenues where new drugs can be developed in an effort to find a cure for the disease.

### Conflict of interest statement

The authors declare that the research was conducted in the absence of any commercial or financial relationships that could be construed as a potential conflict of interest.
